# Supplementary material for: Pyruvate Dehydrogenase Kinase 1 inhibition mediated oxidative phosphorylation enhancement in cartilage promotes osteoarthritis progression
Source: BMC Musculoskelet Disord. 2023 Jul 20;24:597. doi: 10.1186/s12891-023-06585-6 (PMC10357736; doi:10.1186/s12891-023-06585-6)
Supplement: Supplementary file 1 — Additional file 1. [file 12891_2023_6585_MOESM1_ESM.zip › KEGG.Gsea.1653622432464/KEGG_VASOPRESSIN_REGULATED_WATER_REABSORPTION.html]

Details for gene set KEGG\_VASOPRESSIN\_REGULATED\_WATER\_REABSORPTION[GSEA]

|  || Dataset | 55457\_collapsed\_to\_symbols.cls.cls #OA\_versus\_normal.cls.cls #OA\_versus\_normal\_repos |
| Phenotype | cls.cls#OA\_versus\_normal\_repos |
| Upregulated in class | OA |
| GeneSet | KEGG\_VASOPRESSIN\_REGULATED\_WATER\_REABSORPTION |
| Enrichment Score (ES) | 0.21232907 |
| Normalized Enrichment Score (NES) | 0.8268337 |
| Nominal p-value | 0.74390244 |
| FDR q-value | 0.9165637 |
| FWER p-Value | 1.0 |
Table: GSEA Results Summary

  

Fig 1: Enrichment plot: KEGG\_VASOPRESSIN\_REGULATED\_WATER\_REABSORPTION      
 Profile of the Running ES Score & Positions of GeneSet Members on the Rank Ordered List

  

| SYMBOL | TITLE | RANK IN GENE LIST | RANK METRIC SCORE | RUNNING ES | CORE ENRICHMENT || 1 | DYNC1I2 | dynein cytoplasmic 1 intermediate chain 2 [Source:HGNC Symbol;Acc:HGNC:2964] | 373 | 0.717 | 0.0345 | Yes |
| 2 | DCTN6 | dynactin subunit 6 [Source:HGNC Symbol;Acc:HGNC:16964] | 755 | 0.580 | 0.0563 | Yes |
| 3 | PRKACB | protein kinase cAMP-activated catalytic subunit beta [Source:HGNC Symbol;Acc:HGNC:9381] | 771 | 0.576 | 0.1061 | Yes |
| 4 | NSF | "N-ethylmaleimide sensitive factor, vesicle fusing ATPase [Source:HGNC Symbol;Acc:HGNC:8016]" | 908 | 0.540 | 0.1435 | Yes |
| 5 | CREB3 | cAMP responsive element binding protein 3 [Source:HGNC Symbol;Acc:HGNC:2347] | 1036 | 0.510 | 0.1788 | Yes |
| 6 | RAB11A | "RAB11A, member RAS oncogene family [Source:HGNC Symbol;Acc:HGNC:9760]" | 1447 | 0.436 | 0.1855 | Yes |
| 7 | DYNLL1 | dynein light chain LC8-type 1 [Source:HGNC Symbol;Acc:HGNC:15476] | 1578 | 0.416 | 0.2123 | Yes |
| 8 | DCTN4 | dynactin subunit 4 [Source:HGNC Symbol;Acc:HGNC:15518] | 2143 | 0.345 | 0.1990 | No |
| 9 | DCTN2 | dynactin subunit 2 [Source:HGNC Symbol;Acc:HGNC:2712] | 2888 | 0.262 | 0.1644 | No |
| 10 | AQP4 | aquaporin 4 [Source:HGNC Symbol;Acc:HGNC:637] | 2923 | 0.258 | 0.1846 | No |
| 11 | DYNC2LI1 | dynein cytoplasmic 2 light intermediate chain 1 [Source:HGNC Symbol;Acc:HGNC:24595] | 3422 | 0.209 | 0.1644 | No |
| 12 | DCTN1 | dynactin subunit 1 [Source:HGNC Symbol;Acc:HGNC:2711] | 3428 | 0.209 | 0.1825 | No |
| 13 | RAB5C | "RAB5C, member RAS oncogene family [Source:HGNC Symbol;Acc:HGNC:9785]" | 3433 | 0.208 | 0.2006 | No |
| 14 | DYNC1LI1 | dynein cytoplasmic 1 light intermediate chain 1 [Source:HGNC Symbol;Acc:HGNC:18745] | 3773 | 0.179 | 0.1901 | No |
| 15 | AQP3 | aquaporin 3 (Gill blood group) [Source:HGNC Symbol;Acc:HGNC:636] | 3805 | 0.176 | 0.2033 | No |
| 16 | ARHGDIB | Rho GDP dissociation inhibitor beta [Source:HGNC Symbol;Acc:HGNC:679] | 4085 | 0.147 | 0.1947 | No |
| 17 | DYNC2H1 | dynein cytoplasmic 2 heavy chain 1 [Source:HGNC Symbol;Acc:HGNC:2962] | 5033 | 0.070 | 0.1273 | No |
| 18 | ADCY9 | adenylate cyclase 9 [Source:HGNC Symbol;Acc:HGNC:240] | 5191 | 0.057 | 0.1201 | No |
| 19 | DYNC1I1 | dynein cytoplasmic 1 intermediate chain 1 [Source:HGNC Symbol;Acc:HGNC:2963] | 5450 | 0.037 | 0.1033 | No |
| 20 | RAB5B | "RAB5B, member RAS oncogene family [Source:HGNC Symbol;Acc:HGNC:9784]" | 6375 | -0.033 | 0.0345 | No |
| 21 | ARHGDIA | Rho GDP dissociation inhibitor alpha [Source:HGNC Symbol;Acc:HGNC:678] | 6664 | -0.053 | 0.0168 | No |
| 22 | AVP | arginine vasopressin [Source:HGNC Symbol;Acc:HGNC:894] | 7201 | -0.096 | -0.0164 | No |
| 23 | RAB11B | "RAB11B, member RAS oncogene family [Source:HGNC Symbol;Acc:HGNC:9761]" | 7227 | -0.097 | -0.0097 | No |
| 24 | CREB3L1 | cAMP responsive element binding protein 3 like 1 [Source:HGNC Symbol;Acc:HGNC:18856] | 7324 | -0.104 | -0.0079 | No |
| 25 | CREB1 | cAMP responsive element binding protein 1 [Source:HGNC Symbol;Acc:HGNC:2345] | 7470 | -0.116 | -0.0089 | No |
| 26 | CREB3L2 | cAMP responsive element binding protein 3 like 2 [Source:HGNC Symbol;Acc:HGNC:23720] | 7580 | -0.125 | -0.0063 | No |
| 27 | RAB5A | "RAB5A, member RAS oncogene family [Source:HGNC Symbol;Acc:HGNC:9783]" | 8049 | -0.159 | -0.0286 | No |
| 28 | DYNC1H1 | dynein cytoplasmic 1 heavy chain 1 [Source:HGNC Symbol;Acc:HGNC:2961] | 8391 | -0.185 | -0.0387 | No |
| 29 | ADCY3 | adenylate cyclase 3 [Source:HGNC Symbol;Acc:HGNC:234] | 8572 | -0.199 | -0.0350 | No |
| 30 | AQP2 | aquaporin 2 [Source:HGNC Symbol;Acc:HGNC:634] | 8697 | -0.209 | -0.0262 | No |
| 31 | GNAS | GNAS complex locus [Source:HGNC Symbol;Acc:HGNC:4392] | 8929 | -0.226 | -0.0241 | No |
| 32 | VAMP2 | vesicle associated membrane protein 2 [Source:HGNC Symbol;Acc:HGNC:12643] | 8932 | -0.226 | -0.0042 | No |
| 33 | STX4 | syntaxin 4 [Source:HGNC Symbol;Acc:HGNC:11439] | 9222 | -0.250 | -0.0045 | No |
| 34 | DCTN5 | dynactin subunit 5 [Source:HGNC Symbol;Acc:HGNC:24594] | 9366 | -0.261 | 0.0075 | No |
| 35 | CREB5 | cAMP responsive element binding protein 5 [Source:HGNC Symbol;Acc:HGNC:16844] | 9555 | -0.279 | 0.0176 | No |
| 36 | DYNC1LI2 | dynein cytoplasmic 1 light intermediate chain 2 [Source:HGNC Symbol;Acc:HGNC:2966] | 10371 | -0.358 | -0.0140 | No |
| 37 | PRKACG | protein kinase cAMP-activated catalytic subunit gamma [Source:HGNC Symbol;Acc:HGNC:9382] | 10466 | -0.370 | 0.0114 | No |
| 38 | PRKACA | protein kinase cAMP-activated catalytic subunit alpha [Source:HGNC Symbol;Acc:HGNC:9380] | 10611 | -0.384 | 0.0343 | No |
| 39 | AVPR2 | arginine vasopressin receptor 2 [Source:HGNC Symbol;Acc:HGNC:897] | 11512 | -0.502 | 0.0088 | No |
| 40 | PRKX | protein kinase X-linked [Source:HGNC Symbol;Acc:HGNC:9441] | 11634 | -0.522 | 0.0457 | No |
| 41 | ADCY6 | adenylate cyclase 6 [Source:HGNC Symbol;Acc:HGNC:237] | 12005 | -0.594 | 0.0696 | No |
Table: GSEA details [plain text format]

  

Fig 2: KEGG\_VASOPRESSIN\_REGULATED\_WATER\_REABSORPTION      
 Blue-Pink O' Gram in the Space of the Analyzed GeneSet

  

Fig 3: KEGG\_VASOPRESSIN\_REGULATED\_WATER\_REABSORPTION: Random ES distribution      
 Gene set null distribution of ES for **KEGG\_VASOPRESSIN\_REGULATED\_WATER\_REABSORPTION**
